# Misspelled logotypes: the hidden threat to brand identity

**DOI:** 10.1038/s41598-023-45213-0

**Published:** 2023-10-19

**Authors:** Francisco Rocabado, Manuel Perea, Jon Andoni Duñabeitia

**Affiliations:** 1 Department of Education, Universidad Nebrija, 28015 Madrid, Spain; 2 Department of Methodology and ERI-Lectura, Universitat de València, 46010 Valencia, Spain; 3 Department of Language and Culture, The Arctic University of Norway, 9037 Tromsø, Norway

**Keywords:** Psychology, Human behaviour

## Abstract

Brand names are valuable company assets often accompanied by a unique graphical composition (i.e., as logotypes). Recent research has demonstrated that this uniqueness makes brand names and logotypes susceptible to counterfeiting through misspelling by transposition in tasks that require participants to identify correct spellings. However, our understanding of how brand names are incidentally processed when presented as logotypes is incomplete. To address this gap in knowledge, we conducted a virtual reality experiment to explore the transposed-letter confusability effect on brand name recognition. Participants were immersed in a virtual reality setting and incidentally exposed to logotypes that had correctly spelled brand names or included letter transpositions. Offline analyses revealed that participants were more accurate at recognizing brand names that had been presented with correct spellings than those that had been misspelled. Furthermore, participants exhibited false memories for misspelled logotypes, recalling them as if they had been spelled correctly. Thus, our findings revealed that the incidental processing of misspelled logotypes (e.g., SASMUNG) affects the accuracy of logotype identity recognition, thereby underscoring the challenges faced by individuals when identifying brand names and the elements that make counterfeits so effective.

## Introduction

Logotypes are created through a combination of typography (i.e., a specific letter font), color palette, and representative graphic elements (i.e., an image representation of the brand). Logotypes are a primary method for large and small corporations to establish a consistent and uniform visual representation of their brand, enabling easy identification and differentiation from competitors^1^. However, despite the effort put in by big corporates to ensure the consistency of the visual format of their logotypes in an attempt to make their brand memorable when advertising themselves, consumers often fail to identify original brands among counterfeits. A recent European survey revealed that 37% of consumers unintentionally purchased counterfeit goods, with the perceived better quality of counterfeit products being the main deceiving factor and its availability in reputable online marketplaces^2^.

While inspecting the quality of the product might serve as an indicator to detect counterfeit products, the reality is that both conscious and unconscious buying decisions are generally made fast and automatically^3^. Indeed, regular consumers employ only a few seconds to find the product of their need among the variety of alternatives available on the shelf^4,5^. Our study aims to investigate the potential for incidental processing of counterfeit logotypes to lead to misperceptions and false memories. Specifically, we examined the cognitive processes that allow consumers to differentiate between authentic and counterfeit logotypes.

From electronic components to medicines or cosmetics, a common practice for detecting counterfeits involves the inspection of spelling errors^6–8^. In the domain of counterfeit clothing products, strategies such as spelling mistakes (e.g., Valentine instead of Valentino or Calvin Klien instead of Calvin Klein) are frequently employed, rendering the detection of such minor flaws a viable strategy^9^. Yet, this approach might not be as effective as typically presumed. Recent research has revealed that the processes underlying the identification of logotypes share similarities with the processes involved in identifying common words. In tasks that ask participants to indicate whether a logotype was authentic or not, Pathak et al.^10,11^ found the typical benchmark effects in the literature of visual-word recognition, including the key role of the first letter, flexibility of letter identity coding, and, more important for the present paper, flexibility in letter position coding (see^12^ for a recent review). In the field of word recognition, there is a widespread agreement that different manipulations involving the order of internal letters of words are often unnoticed by readers, as compared to changes in the outer (i.e., first or final) letter positions^13^ (see also^14,15^ for reviews). In this line, transposing two middle letters (e.g., judge instead of jugde; cholocate instead of chocolate) are much more prone to confusion with their corresponding base words than control words with replaced letters like jupte or chotonate^12,14^. This later phenomenon is known as the "transposed-letter confusability effect” (see^14,16^).

Recent research has also demonstrated that letter-transposition confusability effects are particularly salient with misspelled logotypes: when participants are asked whether the logotypes’s brand names were correctly spelled, the transposed-letter misspelling amzaon produced much longer response times and more errors than the control, replacement-letter misspelling amceon^17^. Importantly, Perea et al.^17^ also observed a greater magnitude of the transposed-letter effect when brand names were presented within their respective logotypes than in plain text using a non-logotype font. They claimed that this boost in the size of the transposed-letter effect for misspelled logotypes was due to the inherent characteristics of logotypes: logotypes are typically consistent across contexts, featuring a specific and recognizable letter case, typeface, and color combination. Perea et al.^18^ (see also^17^) proposed that during the processing of logotypes, readers encode abstract orthographic information (i.e., letter identity and order) and information arising from the graphical design of the brand name. While the logotypes’ graphical information may speed up their identification, it also makes it more difficult to distinguish the real logotype from a counterfeit created by misspelling (e.g., amzaon with the graphical information of amazon).

Before presenting the logic of the present experiment, it is important to stress that when it comes to encoding and storing new information, humans have been found to exhibit remarkable efficiency and accuracy in their visual memory^19,20^. However, our capacity to recall and recognize graphical elements, particularly symbols incorporated into logotypes, is considerably more limited. These symbols typically comprise simple, easily recognizable shapes, such as the swoosh symbol in Nike or the arrow from A to Z in amazon’s logo. For instance, in two separate experiments, Blake et al.^21^ demonstrated that a mere 10% of participants could accurately reproduce Apple’s logo. Moreover, when presented with a set of distractors in which some aspects of the symbol had been altered (such as the location of the bite in the apple), only 47% of participants could identify Apple’s logomark. This phenomenon can be accounted for by attentional models, which propose that extended exposure to logotypes may result in “attentional saturation” or habituation^21,22^. Consequently, the logotypes’ widespread availability and apparent simplicity can cause inattention amnesia^23,24^. Thus, the reconstructive nature of human memory may allow representative information to intrude due to distilling repeated patterns in our environment. For instance, the recall of Apple’s logomark may begin with a gist-based schema of a real apple, which can interfere with accurately depicting the logomark^21,25,26^.

When dealing with degraded or ambiguous stimuli, it is important to note that individuals can effectively process these word stimuli, even when the structure of the base word has undergone significant alteration (e.g., M473R14L for the word MATERIAL; see^27^). This phenomenon is typically attributed to top-down contextual cues’ role in facilitating stimuli’s legibility^28–30^. An astonishing example of how contextual cues aid recognition happens during children’s literacy emergence through the interaction with environmental prints (e.g., logotypes, labels, road signs). Before learning to read, young children can recognize popular logotypes, but they struggle to identify them when presented in context-free, plain format^31,32^. However, there are times were this type of processing can lead participants to report erroneously seeing words instead of visually similar pseudowords^33^. Contextual information, semantic properties (e.g., shared conceptual and taxonomic features), structural properties (e.g., orthographic or phonological features), and top-down processing significantly impact how individuals perceive elements and events. Such factors can occasionally result in the formation of false memories^34–37^.

In the present experiment, we examined the incidental processing of logotypes (both correctly spelled and misspelled) in forming false memories by engaging participants in a highly immersive virtual reality (VR) setting. It is important to note that Pathak et al.^10^ and Perea et al.^17^ utilized an explicit spelling task wherein participants were specifically instructed to differentiate between authentic and counterfeit (misspelled) logotypes. In contrast, here, we chose a more ecological scenario via the incidental processing of logotypes. Compared to traditional experimental settings, the immersive nature of VR allows for the creation of equally controlled scenarios where participants can be exposed to the stimuli without compromising experimental control. This task required the completion of an orthogonal, independent task (namely, a visual search task) while being incidentally exposed to both correctly spelled and misspelled brand names represented within their respective logotypes. This visual search task entailed counting the number of stars scattered across a VR environment as they navigated through it. They were encouraged to explore the entire VR environment by rotating and shifting their positions to locate and count as many stars as possible. Concurrently, the environment featured various canvas displays showcasing corporate logotypes, providing incidental exposure to the participants. Following a distractor task, aimed at mitigating immediate recall, participants transitioned to a recognition task. This task included a mix of both correctly and incorrectly spelled logotypes. This recognition task assessed the extent of accurate logotype recognition and the potential influence of the earlier incidental exposure within the VR setting.

Given the relatively static graphical nature of logotypes and the considerable potential for confusion arising from misspelled logotypes^17^, we posited that incidental processing of misspelled logotypes, formed by transposing two internal letters, would result in recognition confusion, the generation of false memories, and increased vulnerability to counterfeit reproductions of well-known logotypes. We measured false recognition rates using an old-new recognition task associated with incidental processing of logotypes after an orthogonal task (i.e., counting stars within a virtual reality setting).

To test these hypotheses, we examined three research questions. In the first question, we tested whether the logotypes correctly spelled in the exposure phase would be recognized better in the test phase than those that were misspelled by transposition when presented with the same spelling as in the exposure phase (i.e., “old” items). The logic was that if the misspelled logotypes in the incidental exposure phase were encoded as the original ones (e.g., SASMUNG being identified as SAMSUNG), we would expect lower accuracy scores for the misspellings of logos (i.e., participants may have thought of having seen SAMSUNG in the exposure phase), thus leading to a transposed-letter confusability effect from a mnemonic perspective. Importantly, previous research has shown that the word recognition system eventually assigns letters to positions (i.e., if not, we could not distinguish calm and clam; see^38,39^ for models of letter position coding during visual word recognition). Importantly, our design allowed us to measure the time course of the transposed-letter effect in logotypes by adding the visualization times in the incidental exposure task as a predictor. Thus, we expected the differences in accuracy rates for the “old” logotypes to decrease in magnitude as a function of visualization time (i.e., when participants look at SASMUNG for some time, they may realize that it is a misspelling, thus leading to better recognition rates in the second phase).

In the second question, we focused on the comparison between completely new logotypes (i.e., logotypes that were not present in the exposure phase, regardless of whether they were correctly spelled or not) and their (related) counterparts (i.e., misspellings by transposition in the test phase when they were intact in the exposure phase, or correctly spelled in the test phase when they were misspelled in the exposure phase). The rationale for this comparison is two-fold. On the one hand, we examined the potential increase of false recognition induced by the related logotypes compared to the completely new ones. On the other hand, this comparison allows us to test whether the spelling of the logotypes modulated the above effect in the exposure phase (i.e., whether correctly spelled or misspelled).

Lastly, in the third question, we focused on the recognition rates for those logotypes that had appeared in the exposure phase (i.e., “old” logotypes, either correctly spelled or not) and their related counterparts (i.e., misspelled logotypes in the test phase [e.g., SASMUNG] when the logotypes were intact in the exposure phase [e.g., SAMSUNG] or correctly written logotypes in the test phase when the logotypes were misspelled in the exposure phase). We predicted that incidental processing of counterfeit brand names during exposure, particularly those with transposed letters, would lead to a false memory effect for the original, correctly written versions of those brand names. The logic is that logotypes like SASMUNG may have been encoded as SAMSUNG in the initial exposure phase, given the similarities between the stimuli (see^10,11,17^). Thus, the participants could experience some uncertainty on whether a given stimulus had been presented during the exposure phase, thus yielding a false memory effect for the correctly spelled logotypes.

All in all, this research pioneers a nuanced understanding of visual stimulus recognition by exploring the impact of orthographic manipulations of logotypes on the ability of readers to detect counterfeit products in a real-life scenario. It sheds light on the intrusive effects of false memory creation of counterfeit logotypes and provides insights into the potential dangers of misspelling brand names for counterfeiting. Thus, the present experiment bridges a significant gap in the literature, propelling research on visual word recognition and its implications in real-world settings such as brand recognition and advertising. Finally, should our predictions be confirmed, these results would highlight the need for greater awareness and protection against counterfeit logotypes.

## Methods

### Participants

Participants were recruited from the Universitat de València and Universitat Politècnica de València, who voluntarily participated in the study. The initial participant sample consisted of 30 Spanish undergraduates, but data from 1 participant were not recorded for technical problems. Hence, the final sample consisted of 29 native Spanish university students (17 males with a mean age of 21.9 years old, SD = 3.20, and 12 females with a mean age of 23.3 years old, SD = 3.96), with normal or corrected-to-normal vision. Before beginning with the data collection, all participants signed an informed consent form. This research received full ethical approval from the Research Ethics Committee at Universitat de València (reference 1894511). The experiment was performed in accordance with relevant guidelines and regulations, and the protocol adhered to the Declaration of Helsinki.

### Materials

We selected 16 logotypes of well-known brands among the Spanish population. This set consisted of the stimuli used by Perea et al.^17^ (experiment 2): amazon, SAMSUNG, LACOSTE, Heineken, Levi’s, Colgate, Google, BURGER KING, FANTA, intel, Movistar, Estrella Galicia, MERCADONA, pepsi, and Vodafone. Only brand name logotypes that had a consistent graphical representation on the market and were represented by a combination of lettering style/color/layout, and accompanied by graphical information were eligible (see^17^, for details).

The original letter case of each brand name in the logo, whether uppercase, lowercase, or mixed-case, was maintained (see^40^, for the role of letter case in brand names), for the creation of different stimuli. A transposed letter (TL) misspelled version for each brand name was also created by swapping the order of two adjacent internal letters (e.g., SASMUNG for SAMSUNG). This manipulation was chosen given that preceding word recognition studies have shown that swapping two adjacent internal letters in a word causes more confusion compared to other manipulations (see^14,16^). Moreover, none of the manipulations involved two vowels, given the greater perceptual similarity observed in consonant-transposed than vowel-transposed nonwords^41^. Thus, a total of 32 brand pictures were used in the main experimental phase (i.e., 16 logotypes with the correct spelling and 16 logotypes with letter transpositions).

Furthermore, and for evaluation purposes, eight new brand identities were included in the experimental material of the test phase: McDonald’s, ASTURIANA, IBERDROLA, PRÉSIDENT, HACENDADO, NESCAFÉ, Campofrío, Cruzcampo. The new brand identities were also familiar to the target population. The same orthographic manipulation was applied to them, resulting in 16 logotypes (i.e., eight original logotypes and eight versions with transposed letters).

### Virtual reality materials

**Figure 1:**
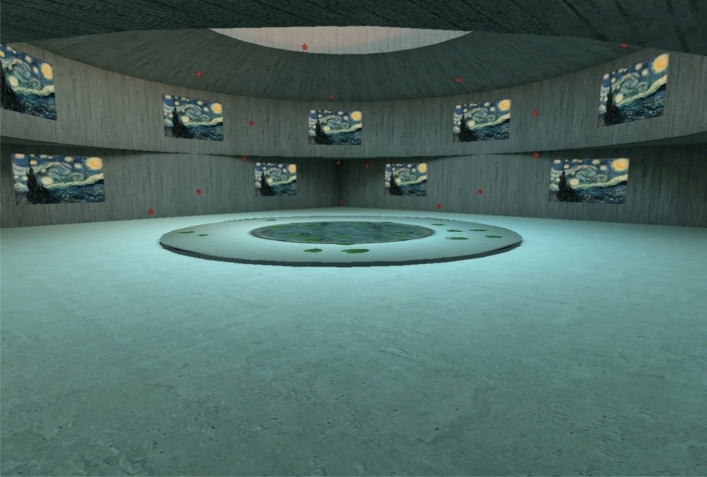
View of the main model presentation and object distribution across the walls and ceiling. Note that canvas textures are included on the figure to represent its position and size dimensions within the gallery.

The stimuli were presented in a virtual reality setting using a head-mounted display (HMD). A main 3D building model acquired on Sketchfab was downloaded to compose our experimental scenario. This main scenario consisted of a 3D 787.40 m × 329.58 m × 787.40 m brutalist gallery model composed of three structural components: a quadrilateral shape layer at base level walls, topped by a circular shape wall and an open dome ceiling (see Fig. 1). Sixteen canvas models of 116.05 m × 72.84 m × 0.81 m were harmoniously spread on the walls of the gallery. These canvas models were used to display the logotypes. Moreover, 40 red stars of 12.52 m × 11.91 m × 1.78 m were randomly distributed over the different walls and ceilings. All model editions were made with Blender^42^. Finally, to improve participants’ immersive Ness in the virtual reality environment, an HDRi background and lighting were included.

### Apparatus

The virtual reality task was programmed with Vizard 6, Python 2.7-based^43^. To ensure quality presentation, stimuli presentation was run on a high-end gaming laptop computer. 3D environment and experimental material were presented through HTC VIVE Pro HMD^44^ at 2880 × 1600 pixel resolution (1440 × 1600 per eye) at a 90-Hz screen refresh rate and a field of view of 110°. Eye-tracking data were collected from the areas of interest from both eyes through the built-in Tobii eye-tracker at a sampling frequency of 120 Hz. The participants’ camera position (i.e., participants’ point of view) within the VR environment was fixed, allowing participants to explore their surroundings by rotating on themselves always from the same still position.

### Task and procedure

The experimental session consisted of an exposure phase, a distractor task, and a test phase. The exposure phase took place in VR to facilitate incidental processing of the experimental material. The materials used by Perea et al.^17^ were divided into two different lists. Each list contained 16 different logotypes, 8 in their original format and the other 8 with the letter-transposition manipulation. The allocation of the logotypes in each list was counterbalanced, so the same logotype never appeared in the two versions in the same list. Participants were randomly assigned to each list. Once the headset was placed and calibrated, participants were given two controllers, which, during the VR immersion, would turn into visible virtual hands. During the exposure phase, participants were instructed to explore their surroundings in search of all the red stars spread across the scenario for just one minute. They were asked to count the exact number of stars spread on the walls while disregarding any other piece of visual information they may encounter. The beginning of the exposure phase (namely, the 1-minute block) was self-paced and started with the display of the logotypes on the canvases and the stars when participants’ pressed one of the controllers. Both canvas and red star locations were kept constant across participants. All 16 canvases were coded as regions of interest (ROI) to assess the time participants spent fixating on the logotypes (counting as valid those fixations that were equal to or larger than 80 ms).

The distractor phase was completed outside VR on a 15.6 inches’ computer laptop and consisted of three different unrelated tasks that lasted 10 minutes. These tasks consisted of a battery of executive functioning assessments.

After the distractor tasks, participants were informed of a final task they had to complete and of which they had not been previously informed. This evaluation phase consisted of a recognition task where a total of 48 brand logotypes were presented. The 16 pairs of logotypes from Perea et al.^17^ in the original and transposed-letter versions, and eight pairs of new well-known logotypes also in the original and transposed-letter versions. Participants were asked to determine whether each of the 48 items had been “seen” or “not seen” during the exposure phase. The instructions were: In this task, you will be presented with 48 images. Using the F and J keys, you will indicate whether each image was present in the first part of the experimental session where you had to count the stars. Press J if the image was present in the first part of the experiment and F if it was not. Please try to respond as quickly and accurately as possible. We recommend keeping your index fingers on the keys throughout the task. These 48 stimuli were divided into three possible main experimental categories: Old (i.e., a logotype presented during the exposure phase in that same format), Related (i.e., a version of the logotype that was not explicitly presented in the exposure phase in that given format), and Unrelated (i.e., a stimuli identity that had not been presented in any format during the exposure phase).

Every trial of the recognition task consisted of a central fixation point (+) presented for 500 ms, immediately followed by the target stimulus presented until a response was given or for a maximum of 4000 ms. A 1000-ms blank screen was included as an inter-trial stimulus. All items were presented in a randomized order, and the presentation and data collection were carried out using Gorilla Experiment Builder^45^.

## Results

Accuracy in the recognition task of the evaluation phase was preprocessed and analyzed using RStudio^46^ in R^47^. Timed-out trials were removed from the accuracy analysis. Descriptive statistics of Accuracy are reported in Table 1. Eye-tracking data corresponding to the summed viewing time of each ROI were also computed and incorporated for further analysis (namely, the Total View Time).

**Table 1:** Mean accuracy rates and standard deviations (in parentheses) across conditions.

Task category	Original format	Transposed-letter format
Old	0.665 (0.47)	0.498 (0.50)
Related	0.443 (0.50)	0.623 (0.50)
Unrelated	0.793 (0.41)	0.867 (0.34)

The data were analyzed using Generalized Mixed Effects Models with the lme4 package^48^. For each of the three research questions, we created different models. The first model included the Logotype Manipulation as a fixed-effect factor (two levels: original [correctly spelled] logotype versus transposed-letter [misspelled] logo) and the Total View Time for items that were incidentally presented during the exposure phase (i.e., Old items) as a covariate. The second model included the same Logotype Manipulation as a fixed-effect factor and the Degree of Relatedness (two levels: related versus unrelated) for items that were seen only once during the evaluation phase. (Of note, although these logotypes were seen only once, related items correspond to their counterpart logotypes that were not seen during the exposure phase but that have a very high visual similarity with the seen ones). Finally, the last model included two fixed-effect factors: Logotype Manipulation (two levels: original [correctly spelled] logotype versus transposed-letter [misspelled] logo) and Degree of Relatedness (old logotypes versus new related logotypes), meaning that the items included on the analysis were the ones seen during the exposure phase and their related counterparts presented during the evaluation phase.

For all questions, we first built the most complex model, and next, the best-fitting model structure was chosen through an automatized model selection process using the dredge function from the MuMln package^49^. This automatization, following an iterative process, compared all the possible model structure combinations of effects and interactions by their goodness-of-fit using the Akaike Information Criterion (AIC). From there, the best model to explain participants’ accuracy scores was obtained and analyzed in jamovi^50^ using the GAMLj module^51^.

### Confusability effects on the recognition of old items

**Figure 2:**
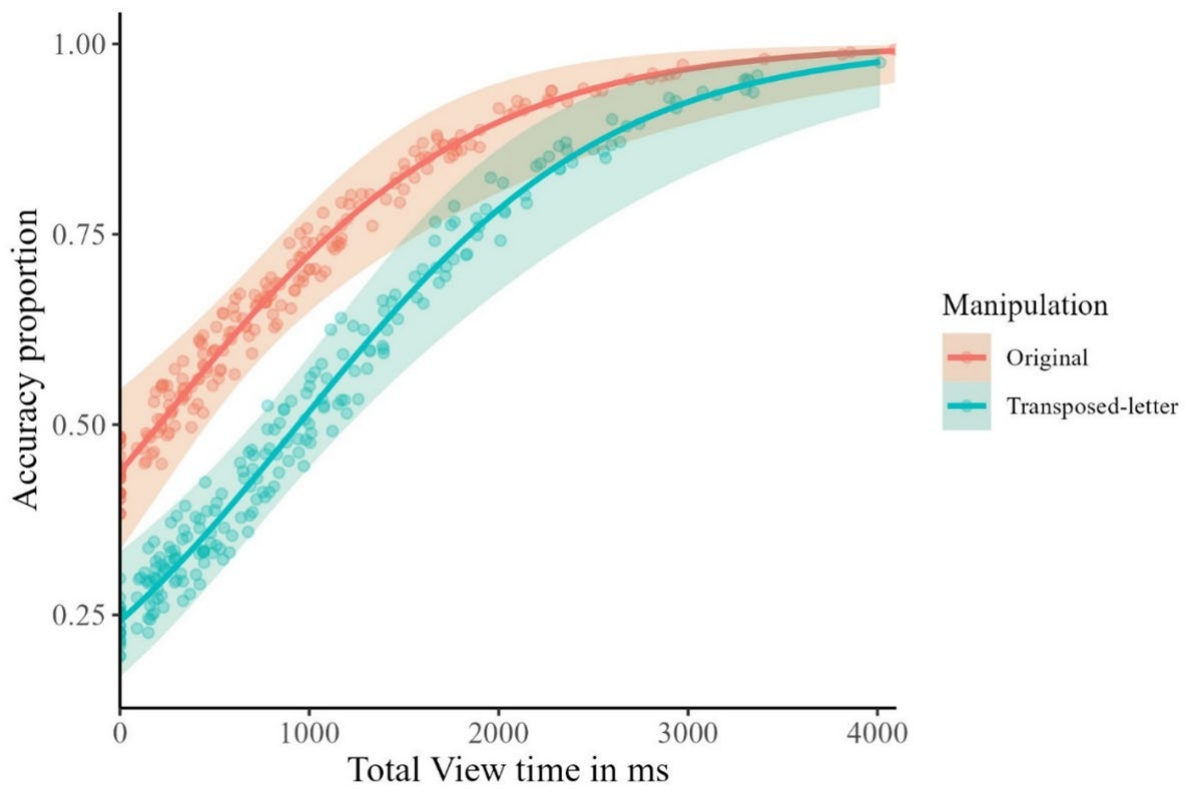
Recognition accuracy scores for incidentally seen logotypes. The blue line represents the original format logotypes and the orange for the transposed letter manipulation. Semi-transparent areas indicate the 95% confidence intervals of the fixed effect of Logotype manipulation.

The best fitting model explaining the accuracy scores for the Old items (i.e., the correct recognition as “seen” of original or transposed-letter logotypes presented during the exposure phase) included the factor of Logotype Manipulation (original vs. transposed-letter) as a fixed effect. Total View Time variable was centered and included as a predictor. Subject and Item were included as random effects. Accurate recognition rates were higher for incidentally seen logotypes in their original format than in their transposed-letter format, χ2(1) = 13.5, *p *< 0.001 (see Fig. 2). In other words, participants were less accurate in identifying previously seen items that were presented with a letter transposition than correctly written brand names (*z* = − 3.67). Moreover, the effect of Total View Time was also significant, χ^2^(1) = 54.0, *p *< 0.001, showing that items that were incidentally fixated for longer times involved better recognition accuracy (*z* = 7.35). Notably, as shown in Fig. 2, prolonged viewing times resulted in near-perfect recognition accuracy for intact logotypes and, to a slightly lesser extent, for misspelled logotypes.

### False memory effects analysis for related versus unrelated items

**Figure 3:**
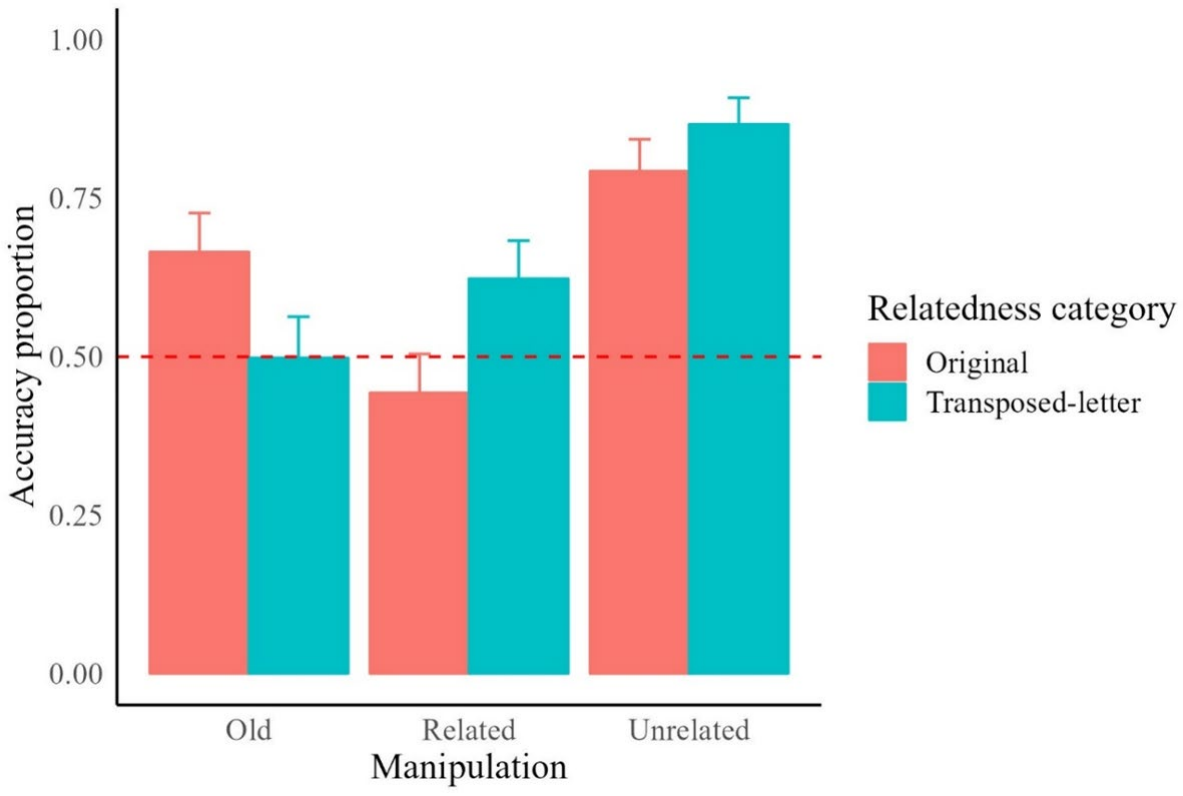
Recognition accuracy scores of seen and unseen logotypes. Bars indicate the 95% confidence intervals of the fixed effect of the relatedness. Dashed lines mark the chance level threshold, above it participants’ responses were given with a certain level of certainty.

The second analysis examined the confusability effects of items that were not presented in the original format. This analysis aimed to explore the degree of similarity between items in the related condition (items not previously seen but presented in the manipulated format during the exposure phase (i.e., misspelled in the exposure phase and correctly written in the test phase or vice versa) compared to the unrelated condition (i.e., completely new logotypes; half correctly written, half with a misspelling by letter transposition). The best-fitting model for accuracy scores included Relatedness Category (related or unrelated) and Logotype Manipulation (original or misspelled via letter transposition) as fixed effects. Subject and Item were included as random effects. Accuracy recognition rates were significantly higher for items that were never presented before, χ^2^(1) = 65.05, *z* = 8.06, *p* < 0.001. Moreover, logotypes presented in a transposed-letter format showed higher accuracy rates than logotypes displayed in their original format, χ^2^(1) = 12, *z* = 3.46, *p *< 0.001 (see Fig. 3). Finally, no significant interaction was found between Relatedness Category and Logotype Manipulation, χ^2^(1) = 0.33, *z* = − 0.58, *p* = 0.56. This analysis revealed two important findings. Firstly, participants could accurately identify logotypes unrelated to any previously seen items and contained misspelled brand names as never seen. Secondly, participants performed at chance level when identifying correctly spelled brand names presented in a transposed-letter counterfeit version during the exposure phase, highlighting the creation of false memories for such manipulated versions.

### Orthographic effects on the recognition of old versus related items

This third analysis further examined the accuracy of recognition scores between old, seen items and related, unseen items. The model for accuracy included the fixed effects of Relatedness Category (old vs. related) and Logotype Manipulation (original vs. transposed-letter), and the random effects of Subject and Item. Accuracy recognition rates were slightly higher for incidentally seen logotypes than for unseen logotypes, but this difference was not significant χ^2^(1) = 3.08, *z* = 1.76, *p* = 0.079. There were no significant differences in recognition accuracy between logotypes presented in their original form and those with transposed letters, χ^2^(1) = 0.01, *z* = 0.13, *p* = 0.898. More importantly, we found a significant interaction between Logotype Manipulation and Relatedness Category, χ^2^(1) = 29.32, *z* = − 5.42, *p *< 0.001 (see Fig. 3).

Simple effect tests showed that accuracy rates for old logotypes (namely, those logotypes seen during the incidental exposure phase) with a letter transposition (i.e., misspelled logotypes) were significantly lower compared to logotypes presented in their original format, χ^2^(1) = 11.7, *z* = − 3.43, *p* < 0.001). Conversely, related unseen logotypes in their original format were more difficult to identify as not previously seen compared to logotypes with the transposition manipulation (χ^2^(1) = 14.4, *z* = 3.80, *p* < 0.001). Furthermore, as can be seen in Fig. 3, when an item was presented in the exposure phase with a letter transposition, participants performed at chance level when deciding whether they had previously seen this item and its corresponding original version.

## Discussion

The present experiment, conducted in a virtual reality setting, examined the type of memory representations formed during the incidental processing of logotypes. Firstly, we found that misspelled logotypes were less accurately recognized as seen logotypes than correctly spelled ones. This indicates that, even under an incidental scenario, participants encode letter position correctly (i.e., when encountering a misspelled logotype, participants encode it as genuine). From a mnemonic perspective, these results generalize the transposed-letter confusability effect observed in previous research where participants initially tend to encode CHOLOCATE as its base word CHOCOLATE (e.g., see^16^; see also^15^). Secondly, the duration of visualization played a crucial role in determining the accuracy of recognizing previously viewed logotypes. Longer viewing times in the exposure phase not only increased the accuracy rates not only of genuine logotypes but also of misspelled logotypes. Indeed, the accuracy rates of misspelled logotypes (i.e., recognizing a misspelled logotype in the exposure phase as a misspelling in the test phase) tend to be comparable to those in their original versions as a function of viewing time in the exposure phase. This finding supports the idea that letter position tends to be more accurate with time (see^39^, for a model of letter position coding in which the uncertainty when assigning positions to letters is progressively resolved). Thirdly, exposure to manipulated logotype identities disrupted the perception of related yet unseen brand logotypes, leading to high levels of uncertainty during recognition testing. And lastly, incidental exposure to counterfeit logotypes resulted in the formation of false memories for correctly spelled brand names (i.e., SASMUNG was often encoded in the exposure phase as SAMSUNG).

Building on the aforementioned findings, our study further unveils that minor orthographic alterations significantly modulate the ability to accurately remember logotype identities. Specifically, the inclusion of transposed-letter manipulations during incidental processing (e.g., SASMUNG instead of SAMSUNG) resulted in a high level of uncertainty in logotype recognition, which in turn increased the rate of false recognition. These findings extend the current literature on human memory’s susceptibility to logotype representations, likely driven by incidental exposure to manipulated (counterfeit) logotype identities that bear a perceptual resemblance, albeit not identical to the originals. Therefore, our ability to remember accurate logotype identities can be easily swayed, especially when encountering orthographic manipulations of brand names: a transposed-letter manipulation during incidental processing can lead to false recognition and high levels of uncertainty in logotype recognition. These outcomes have substantial implications in the areas of brand recognition and counterfeiting. They suggest a need for enhanced brand protection strategies to mitigate the risks associated with counterfeit logotypes, which could, in turn, affect brand integrity and consumer trust. Moreover, our study sets the stage for further exploration into the cognitive mechanisms underpinning logotype recognition and the potential interventions to bolster accurate brand recall amidst orthographic manipulations.

Furthermore, beyond recognizing the seen original and unseen transposed-letter logotypes, our findings reveal an intriguing aspect of incidental exposure to counterfeit logotypes elicits false memories for the original correctly spelled brand names. These false memories, in turn, increased the level of uncertainty during the recognition phase, as participants showed difficulties identifying whether they had previously seen logotypes when they were presented with a letter transposition. This is similar to what has been observed with regular words, where misspelled words produce high false recognition rates^30^. The presented study showed that misspelled brand names also yielded high false recognition rates of original brands, where participants recognized an original brand name as seen before when it was actually presented with a transposed-letter manipulation during the exposure phase. These findings support previous research showing that pseudowords created by letter transpositions are perceptually similar to the base words^14,16^ and that processing a transposed-letter string activates the lexical and sub-lexical representation of their base word^52,53^. For example, a string-like relovution activates the base word revolution, facilitating its processing. A similar process occurs for a counterfeit brand name like Relovut, which may activate and facilitate the processing of the brand name Revolut.

Aligning with past research, our results demonstrate that individuals are more likely to falsely recognize misspelled words than correctly spelled words^30,54,55^. Individuals develop precise representations of the word’s spelling, pronunciation, and meaning for commonly encountered words, forming strong connections between these components^56^. Reading printed words leaves memory traces of the word’s orthographic, phonological, and semantic features^57,58^. Misspellings are not merely the result of poor orthographic representations but are integrated into the mental lexicon as surface-level variations of the correct word^29,30,55^. Thus, when encountering a word like receive with various possible spellings, such as recieve, receeve, or resieve, individuals also incorporate these variations into their mental representation of the correct spelling.

At a theoretical level, many influential neurobiologically-based models of visual word recognition propose that attributes like case, color, or font are disregarded during word processing^59,60^. Our findings pose some limits to this assumption, especially in the context of brand names. Previous research on brand name identification^40^ reveals that the case of brand names (uppercase or lowercase) significantly affects their recognition—for instance, ‘adidas’ is recognized quicker than ‘ADIDAS’, and ‘IKEA’ faster than ‘ikea’. Similar findings have been reported in the processing of various letter strings such as acronyms^61^, proper names^62^, German nouns^63^, logotypes^17^, and city names^64^. When these stimuli are presented in their most common format, a measurable advantage is observed (e.g., faster identification times). This poses a challenge to visual-word recognition models that assume a case-invariant abstract letter/word representation, suggesting the possibility of an orthographic lexicon that retains not only the word’s abstract letter units but also specific indexical properties like font or letter case (see^65^, for an episodic theory of lexical access), in particular for those stimuli with a consistent visual format. In this context, visually presented stimuli, when displayed in their archetypical representation or most familiar form, could trigger a higher level of activation of previous episodes in the lexicon. This notion aligns with our findings on the recognition of transposed-letter logotypes and the false memories they induce. Given that most familiar form representation on brand names logos are the result of mainly graphical information one plausible explanation could be that such characteristics trigger bigger level of activation, thus limiting our capacity to attend the pure orthographical information.

On an applied front, with the escalating prevalence of counterfeit goods, there’s a quest for solutions extending beyond significant alterations to the original brand logotype are being sought. Recently, brand logotypes have shifted towards simpler graphical representations, likely due to evidence that visually complex fonts require higher cognitive processing (see^66^). Our study highlights the similarities between reading words and brand names in logotypes, revealing how incidental letter confusability can affect brand recognition and contribute to the success of fake products that manipulate minimal orthographic elements. Unlike the form flexibility of regular word writing, logotypes have constant orthographical forms with minimal variations in their constituent graphical elements, leading to the assumption of stable mental representations. Our findings reveal that a distinct logotype design intended for easy identification comes at an unforeseen cost: a slight alteration in orthography (such as letter transposition resulting in a misspelling) may lead to the perception of a counterfeit logo as the authentic one.

Our findings reveal the intricacies of the transposed-letter confusability effect in brand name identification: consumers may temporarily encode misspelled names of well-known brands as correctly spelled brands. While this might seem to initially enhance brand exposure, it predominantly leads to high false recognition rates, creating confusion and uncertainty among consumers regarding the original brand. This confusion could extend to associating the original brand with incorrect or counterfeit products, which is particularly detrimental when the counterfeit products are of inferior quality compared to the original ones^67^. Clearly, such associations could adversely affect the original brand’s reputation and consumer trust. Moreover, the false memory effect predicted in our study further highlights the negative implications, where consumers exposed to counterfeit brand names with transposed letters could harbor uncertainty regarding the original brand. The cumulative effect of these factors suggests that the negative implications on original brand recognition and identity outweigh the potential benefits of increased brand exposure, underscoring the hidden threat posed by misspelled logotypes to brand identity, especially in a market proliferated by counterfeit goods.

The advent and easier accessibility of Virtual Reality (VR) technology have opened up new avenues for researchers, demonstrating the feasibility and viability of utilizing VR in experimental psychology as a transitional tool from 2D experimentation to a 3D immersive one^68,69^. The present findings, using a novel VR setting during word recognition (see^70^, for recent word recognition research using VR), showed that the difficulty in accurately encoding and recognizing the detailed characteristics of complex logotypes, including their orthographic representations, extends to genuine and counterfeit versions. Despite regular exposure to brand names daily, our ability to accurately identify counterfeit versions is relatively poor when we are incidentally exposed to them, thus making it challenging to distinguish between genuine and fake brand logotypes. Moreover, with advances in advertising where augmented reality billboards are showcasing logotypes in a never-before-seen manner, there is a growing need to further research how these changes in logotype representation benefit brand names’ mental representation as further brand name identities will be created. This emerging trend underscores the importance of our study in understanding the intricate dynamics of visual stimulus recognition and logotype manipulation in modern, technologically advanced settings. Building on prior research, our study emphasizes the poor orthographic processing and visual memory for common objects in naturalistic environments^25,26,71^, contributing to a deeper understanding of both orthographic processes and their interaction with the cognitive and metacognitive mechanisms underlying memory for ecological information in real-world settings^71^. In summary, the present study elucidates the dynamics of visual stimulus recognition and logotype manipulation, laying a robust foundation for future investigations into the real-world implications of these findings, particularly in the domains of consumer behavior and brand protection.

## Data Availability

The datasets used and analysed during the current study are available from the corresponding author on reasonable request.
